# Progestogens to prevent preterm birth in twin pregnancies: an individual participant data meta-analysis of randomized trials

**DOI:** 10.1186/1471-2393-12-13

**Published:** 2012-03-15

**Authors:** Ewoud Schuit, Sarah Stock, Rolf HH Groenwold, Kimberly Maurel, C Andrew Combs, Thomas Garite, Cathy Y Spong, Elizabeth A Thom, Dwight J Rouse, Steve N Caritis, George R Saade, Julia M Zachary, Jane E Norman, Line Rode, Katharina Klein, Ann Tabor, Elçin Çetingöz, John C Morrison, Everett F Magann, Christian M Briery, Vicente Serra, Alfredo Perales, Juan Meseguer, Anwar H Nassar, Arianne C Lim, Karel GM Moons, Anneke Kwee, Ben Willem J Mol

**Affiliations:** 1 Julius Centre for Health Sciences and Primary Care, University Medical Centre Utrecht, Utrecht, the Netherlands; 2 MC Centre for Reproductive Health, University of Edinburgh, Edinburgh, Scotland, USA; 3 The Centre for Research, Education & Quality (CREQ), Obstetrix Medical Group, Fountain Valley, California, USA; 4 Obstetrix Medical Group, San Jose, California, USA; 5 The Centre for Research, Education & Quality (CREQ), Obstetrix Medical Group, Steamboat Springs, Colorado, USA; 6 National Institute of Child Health and Human Development, Bethesda, Maryland, USA; 7 The Biostatistics Centre, George Washington University, Rockville, MD, USA; 8 Department of Obstetrics and Gynecology, Brown University, Providence, Rhode Island and Providence Plantations, USA; 9 Department of Obstetrics and Gynecology, Magee Womens Hospital, University of Pittsburgh, Pittsburgh, USA; 10 Department of Obstetrics and Gynecology, University of Texas Medical Branch, Galveston, USA; 11 Department of Fetal Medicine 4002, Copenhagen University Hospital, Rigshospitalet, Copenhagen, Denmark; 12 Department of Obstetrics and Feto maternal Medicine, Medical University of Vienna, Vienna, Austria; 13 Department of Obstetrics and Gynecology, Zeynep Kamil Women and Children Diseases Education and Research Hospital, Uskudar, Istanbul, Turkey; 14 Department of Obstetrics and Gynecology, University of Mississippi Medical Centre, Jackson, USA; 15 Department of Obstetrics and Gynecology, University of Arkansas for Medical Sciences, Little Rock, Arkansas, USA; 16 Department of Obstetrics and Gynecology, Willis Knighton Medical Centre, Shreveport, Louisiana, USA; 17 Maternal-Fetal Medicine Unit, Instituto Valenciano de Infertilidad, University of Valencia, Valencia, Spain; 18 Department of Obstetrics and Gynecology, University Hospital La Fe, Valencia, Spain; 19 Departamento de Obstetricia y Ginecología, Hospital Universitario Virgen de la Arrixaca, Murcia, Spain; 20 Department of Obstetrics and Gynecology, American University of Beirut, Beirut, Lebanon; 21 Department of Obstetrics and Gynecology, Academic Medical Centre, Amsterdam, the Netherlands; 22 Department of Obstetrics, and Gynecology, University Medical Centre Utrecht, Utrecht, the Netherlands

## Abstract

**Background:**

Preterm birth is the principal factor contributing to adverse outcomes in multiple pregnancies. Randomized controlled trials of progestogens to prevent preterm birth in twin pregnancies have shown no clear benefits. However, individual studies have not had sufficient power to evaluate potential benefits in women at particular high risk of early delivery (for example, women with a previous preterm birth or short cervix) or to determine adverse effects for rare outcomes such as intrauterine death.

**Methods/design:**

We propose an individual participant data meta-analysis of high quality randomized, double-blind, placebo-controlled trials of progestogen treatment in women with a twin pregnancy. The primary outcome will be adverse perinatal outcome (a composite measure of perinatal mortality and significant neonatal morbidity). Missing data will be imputed within each original study, before data of the individual studies are pooled. The effects of 17-hydroxyprogesterone caproate or vaginal progesterone treatment in women with twin pregnancies will be estimated by means of a random effects log-binomial model. Analyses will be adjusted for variables used in stratified randomization as appropriate. Pre-specified subgroup analysis will be performed to explore the effect of progestogen treatment in high-risk groups.

**Discussion:**

Combining individual patient data from different randomized trials has potential to provide valuable, clinically useful information regarding the benefits and potential harms of progestogens in women with twin pregnancy overall and in relevant subgroups.

## Background

### Preterm birth in twins

Preterm birth in multiple pregnancies is a major public health concern. Stillbirths and neonatal deaths in twins are 3 and 6 times higher than in singletons and a disproportionate amount of long-term morbidity is associated with multiple pregnancies [1]. Preterm birth is the principal factor contributing to these adverse outcomes, with 50% of twin pregnancies delivering before 37 weeks and 9% delivering before 32 weeks [2]. Improving outcomes in multiple pregnancies is a goal of modern obstetrics, but as yet, few interventions have been proven to be of benefit in this group. Thirty-three to 56% of preterm births in twins are due to spontaneous preterm labor, making the prevention of preterm labor an attractive strategy [2,3].

### Progestogens to prevent preterm birth

Randomized trials in singleton pregnancies have suggested that antenatal progestogens (including vaginal progesterone and synthetic progestogens such as 17-hydroyprogesterone caproate [17-OHPC]) prevents preterm delivery in women who are at high risk of preterm delivery because of a previous preterm delivery [4,5] or a short cervix [6]. These trials have led investigators to examine whether antenatal progestogens could decrease preterm birth in multiple pregnancies.

Six randomized controlled trials of progestogens to prevent preterm birth in twin pregnancies have now been published, two large trials from the USA [7,8] and one from the UK [9] and three smaller trials from Turkey [10], the UK [6] and the USA [11]. These studies randomized women to either 17-OHPC/vaginal progesterone pessary, and placebo. One study, which included 67 women with twin pregnancies, 100 mg of vaginal progesterone was found to reduce delivery before 37 weeks gestation (OR 3.48 [1.2-10.5]) [10]. In all other studies [6,9,7], treatment with 17-OHPC or vaginal progesterone did not lead to any significant reduction in preterm delivery or fetal loss. In the two largest trials, however, a non-significant increase in intrauterine death was seen in the treatment group [8,9]. Furthermore, there was found a significant difference in median gestational age favoring placebo, in the other large trial [7]. We are aware of five other trials of progestogens in multiple pregnancy that are nearing completion or publication [12-16]. In total these trials have included 3,522 women and more than 7,000 infants. Combining data from these high-quality clinical trials has potential to provide valuable information regarding the benefits and potential harms of progestogens.

### Rationale for an IPD meta-analysis

Aggregated data meta-analysis involves synthesis of estimates from clinical trials. This allows for a more robust estimate of the overall treatment effect of progestogen on multiple pregnancies as well as a more conclusive evaluation of any harmful effects. This is particularly important, as two published RCTs of progestogens in twins have shown a non-significant trend for increased intrauterine death with progestogen treatment [8,9]. A potential problem in aggregated data meta-analyses is that primary outcomes of clinical trials as well as subgroups defined in clinical trials can differ, which makes it impossible to pool the results of different studies. An Individual participant data (IPD) meta-analysis overcomes this problem as it involves synthesis of individual level data from clinical trials. This allows for the same robust estimate of the treatment effect and harmful effects as in aggregated data meta-analysis, only now more flexibility is possible regarding the choice of endpoints, subgroups and potential harms.

Performing an IPD meta-analysis as opposed to an aggregated data meta-analysis has further advantages. Firstly, IPD allows standardization of inclusion and exclusion criteria and analysis across studies, independent of bias that may arise through selective reporting [17]. Secondly, IPD allows for exploration of a differential treatment effect in relevant subgroups (i.e. treatment covariate interactions), for example, women with a monochorionic twin pregnancy and women with a short cervix [18]. Since IPD meta-analyses include more detailed data on a patient level than aggregated data meta-analyses, statistical power to carry out informative subgroup analyses is higher. Furthermore, flexibility of subgroup analyses is enhanced, thus the estimated subgroup effects may be less influenced by misclassification and bias. IPD meta-analysis therefore allows for a valid assessment of differences in treatment effects across subgroups [19]. Thirdly, IPD allows time-to-event analysis. Conventional meta-analysis only allows a pooled estimate of treatment effect at specified cut-points, i.e. delivery before 32, 34 or 37 weeks. The combined analysis of individual data however, can take account of the time between the initiation of treatment and the outcome of interest [20]. This allows time-to-delivery analysis with the construction of Kaplan-Meier analysis and the performance of Cox regression. This means associations between the timing and duration of progestogen treatment and preterm birth and intrauterine death can be explored. This is important because most published trials have reported a non-significant trend towards a shorter duration of pregnancy after the use of progestogens in women with a multiple pregnancy [8,9,21,22].

## Methods/designs

### Criteria for inclusion of studies in IPD

We propose an IPD meta-analysis of randomized controlled trials of 17- OHPC or vaginal progesterone versus placebo in women with twin pregnancies.

### Participants

Inclusion criteria will be women with twin pregnancies, with chorionicity and gestation confirmed by ultrasound, who were included in a RCT comparing progestogens with placebo for the prevention of preterm birth. Women with congenital abnormalities, contraindications to progestogen treatment and cervical cerclage will be excluded.

Although studies have been performed in women with triplet pregnancies [12,21,22] these will not be included in the IPD meta-analysis. There appear to be differences in the response to progestogens in women with singleton and twin pregnancies, therefore further differences might be anticipated between women with twins and those with higher order multiples. As the number of trial participants with triplet pregnancies is comparatively small compared to the number with twin pregnancies, excluding these women should not negatively affect the power of the meta-analysis, whilst ensuring the group is as homogenous as possible. Exclusion of triplet pregnancies from the analyses will not compromise the validity of the study since randomization was stratified for twin or higher order multiple in the studies that included both twin and triplet pregnancies [7,8,12,21,22].

### Intervention

The intervention will be either weekly intramuscular injection of 17-hydroxyprogesterone caproate (17-OHPC) or daily vaginal progesterone. As these treatments may act differently and have different distribution profiles we will analyze the results of the two types of treatment separately.

### Outcome measures

The primary outcome will be adverse perinatal outcome, a composite outcome of perinatal death, defined as death before discharge from the hospital, and significant neonatal morbidity at discharge, defined as one or more of respiratory distress syndrome (RDS) requiring ventilation for ≥ 24 hr, bronchopulmonary dysplasia (BPD), intraventricular hemorrhage (IVH) grade III or IV), periventricular leucomalacia (PVL), necrotizing enterocolitis (NEC) grade II or more) culture proven sepsis, retinopathy of prematurity (ROP) requiring treatment.

Secondary outcomes will be intrauterine death < 32 weeks or preterm birth < 32 weeks; intrauterine death < 35 weeks or preterm birth < 35 weeks; intrauterine death < 37 weeks or preterm birth < 37 weeks; intrauterine death; fetal loss < 28 weeks or early preterm birth < 28 weeks gestation; time to delivery or death. If data is detailed enough, preterm birth will be analyzed separately for spontaneous preterm birth and indicated preterm birth.

### Subgroup analyses

Subgroup analyses will be performed for the primary outcome only, in the following groups

- each of ultrasonographically diagnosed monochorionic and dichorionic twins

- women who completed ≥ 90% of treatment

- women with a cervical length < 25 mm on baseline assessment (in studies where transvaginal cervical length measurement was specified in protocol)

- women with a prior spontaneous preterm birth < 37 weeks

- ethnicity

- each dose of vaginal progesterone, e.g. ≤ 100 mg versus ≥ 200 mg.

### Identification of studies

We will perform an electronic search of the Cochrane Central Register of Controlled Trials (CENTRAL), PubMed, MEDLINE and ClinicalTrials.gov for published or registered randomized controlled trials including women with twin pregnancy that were randomly allocated to treatment with progestogens (including vaginal progesterone and 17-hydroxyprogesterone caproate) or placebo in the second or third trimester with the intention to prevent preterm birth. We will use the search terms "preterm birth" AND ["progesterone" OR "17 hydroxyprogesterone caproate" OR "progestogen"] AND ["pregnancy multiple" OR "pregnancy twin"] AND "randomized controlled trial" AND "human".

Two review authors (ES and SS) will independently assess inclusion criteria and study quality and risk of bias. A third author (BWJM) will review studies in which there is any disagreement about study quality. Risks of bias will be assessed in all of the identified studies based on [23]:

- sequence generation (i.e. computer generated random number, use of random number table or other truly random process)

- allocation concealment (i.e. web-based or telephone central randomization or consecutively numbered sealed opaque envelopes)

- blinding for participants, study personnel and outcome assessors

- Incomplete outcome data

- Selective outcome reporting

- Other sources of bias

In cases where study quality is not clear from trial protocols or publications, then the authors will be contacted for clarification.

The corresponding authors of eligible studies will be approached to take part in the IPD meta-analysis. They will be invited to take part if the study is complete and data available by 1^st ^July 2011.

Data quality will be independently assessed by two review authors (ES and SS). A third author (BWJM) will review data in which there is any disagreement about quality. Only studies with adequate outcome data (< 10% participant attrition or exclusion, with full reporting of reasons for withdrawals and protocol violations and no imbalance in drop-outs across groups) and adequate reporting (all of the study's pre-specified outcomes and all expected outcomes of interest made available) will be included.

### Analysis

Overall effects of each treatment (17-OHPC and vaginal progesterone) in women with twin pregnancies will be estimated in the pooled IPD. Descriptive comparisons between studies will be conducted to assess between-study differences. We assume the data to be missing at random (MAR), therefore observed patient characteristics will be used to impute missing data, by means of multiple imputation [24]. Missing data will be imputed within each original study, before data of the individual studies are pooled. Treatment effects will be estimated by means of a random effects log-binomial model and, hence, the measure of association is the risk ratio. The presence of heterogeneity of outcomes across trials will be assessed using the I^2 ^measure [25]. Heterogeneity across studies and dependency between data originating from the same study will be taken into account by fitting a random intercept for each original study. If necessary, analyses will be adjusted for variables used in stratified randomization. Furthermore, dependency between children born from the same pregnancy will be accounted for by means of generalized estimating equations (GEE) [26]. To investigate subgroup effects, the treatment effects will also be estimated within strata based on single subgrouping variables, as well as using an interaction term in the regression model.

Time-to-delivery analysis will be performed with Kaplan-Meier analysis and Cox proportional hazards regression analysis. Again, dependency between data originating from the same study will be taken into account by conducting a stratified analysis (stratified by study) [27].

We will perform subgroup analysis with pooled individual datasets of women stratified by the pre-specified criteria outlined above. Where available we will plot cervical length against gestational age of cervical length measurement. When differences in gestational age explain differences in length, we will apply standardization for gestational age. Differences in cervical length between the studies will also be explored. We will assess the absolute value of cervical length (corrected for gestational age differences) as well as the percentiles of cervical length (5th, 10th and 25th) in each dataset. We will assess interaction between the treatment effect of progestogens and cervical length, using both time to delivery and the primary and secondary endpoints. To ensure that subgroup effects are not confounded by between-trial differences, dependency between data originating from the same trial will be taken into account using a random intercept for every study in the regression model [18].

## Discussion

The proposed IPD meta-analysis is necessary to determine whether progestogen treatment in twin pregnancy is beneficial or harmful. This is the first study that combines data on the effect of progestogens in twins and the proposed IPD methodology will maximize the impact of results.

The protocol for the individual participant data meta-analysis has been designed with input from the authors of ten randomized controlled trials of progestogens in women with twin pregnancies, many of which have been published in high impact factor journals (Table 1). All authors have committed to providing data if their studies meet inclusion criteria. In total these trials have included 3,498 women and almost 7,000 infants, allowing the meta-analysis to explore effects of progestogens on rare outcomes and in high-risk subgroups. We anticipate it will provide definitive data synthesis guiding clinical practice and future research in this area.

**Table 1:** Overview of the studies published by authors consulted when planning the IPD meta-analysis

Study	Period	N	Intervention	Primary outcome
**Briery**[11]	06/04-06/10	30	250 mg 17-OPHC or placebo	delivery before 35 completed weeks' of gestation

**Cetingoz**[10]	12/04-02/07	67	100 mg vaginal progesterone or placebo	delivery before 37 weeks

**Lim**[12]	08/06-07/09	654	250 mg 17-OHPC in 1 mL castor oil or placebo	composite outcome (Severe RDS, BPD, IVH grade III or worse, NEC, proven sepsis or death before discharge)

**Combs**[7]	11/04-02/10	240	250 mg 17-OHPC or placebo	composite outcome (RDS, Oxygen therapy at 28d, Neonatal sepsis, Pneumonia, IVH grade III or worse, periventricular leukomalacia, NEC, retinopathy of prematurity, asphyxia)

**Nassar**[13]	10/06-10/10	290	250 mg 17-OPHC or placebo	frequency of delivery prior to completed 37 weeks of gestation (259 days)

**Norman**[9]	12/04-04/08	500	Vaginal progesterone gel 90 mg or placebo	delivery or intrauterine death before 34 weeks of gestation

**Rode**[14]	06/06-09/10	650	200 mg vaginal progesterone of placebo	incidence of delivery < 34 weeks

**Rouse**[8]	04/04-02/06	661	250 mg 17-OHPC in 1 mL castor oil or placebo	composite outcome (delivery or fetal death before 35 completed weeks of gestation)

**Rozenberg**[15]	06/06-06/10	160	500 mg 17-OPHC or no treatment	Interval between inclusion and delivery

**Serra**[16]	01/06-05/08	246	200 mg or 400 mg vaginal progesterone or placebo	Preterm birth rate (< 37 weeks)

## Abbreviations

17-OHPC: 17-alphahydroxyprogesterone caproate; GEE: Generalized estimating equations; IPD: Individual participant data; MAR: missing at random; RCT: randomized controlled trial.

## Competing interests

The authors declare that they have no competing interests.

## Authors' contributions

ES, ACL and BWJM were involved in the concept and the design of the study. ES and SS planned and wrote the initial protocol with significant contributions by RHHG, AK, JEN, KGMM, DJR and BWJM. ES, SS, RHHG, KM, CAC, TG, CYS, EAT, DJR, SNC, GRS, JMZ, JEN, LR, AT, JCM, EFM, VS, AP, JM, ACL, KGMM, AK and BWJM participated in face-to-face meetings and/or teleconferences to discuss the protocol, the design of the meta-analysis, the choice of outcome measures and analysis strategies. KM, CAC, TG, CYS, EAT, DJR, SNC, JEN, LR, KK, AT, EC, PR, JCM, EFM, CMB, VS, AP, JM, AHN, ACL, BWJM were involved in the construction and design of one of the trials included in the meta-analysis. All authors critically reviewed the subsequent versions of the manuscript and approved the final manuscript.

## Pre-publication history

The pre-publication history for this paper can be accessed here:

http://www.biomedcentral.com/1471-2393/12/13/prepub
